# Supplementary material for: Functional connectivity of the Precuneus reflects effectiveness of visual restitution training in chronic hemianopia
Source: Neuroimage Clin. 2020 May 26;27:102292. doi: 10.1016/j.nicl.2020.102292 (PMC7303670; doi:10.1016/j.nicl.2020.102292)
Supplement: Supplementary Data 1 [file mmc1.docx]

**Supplementary Material**

Each patient's lesion site was localised using the lesion_gnb toolbox for SPM (Griffis et al., 2016). Of the 20 patients, eight had a lesion in the right hemisphere, and 11 had a lesion in the left hemisphere. One patient had a bilateral lesion. Investigating the overlap between the significant cluster in the left Precuneus and the patients' lesion site showed that the lesion site did not cover the left Precuneus. One patient showed overlap between his or her lesion site and the marginally significant cluster in the right hemisphere. More specifically, in this cluster (285 voxels in total), 60 voxels fell within the patient's lesion site. Figure I shows the marginally significant cluster in the right hemisphere projected on the anatomical image of this patient's brain.


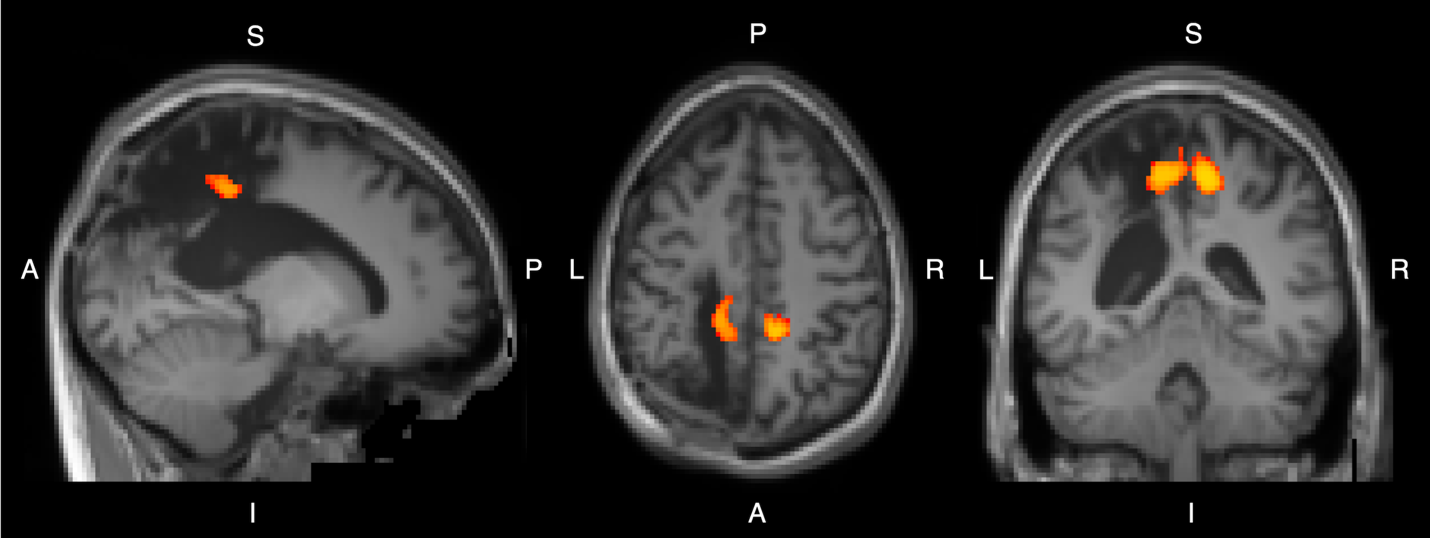
 **FIGURE I | Randomised results partially overlap lesion site in one patient.** The randomise results of the Occipital Pole network and the obtained attention-modulated change in sensitivity, thresholded at *p* < 0.05, projected on the anatomical image of one of the patients (normalised to MNI). Note that the marginally significant cluster in the right hemisphere falls within the patient’s lesion site. From the anatomical image identifying information is removed using mri_deface, the automated defacing tool of Freesurfer (http://surfer.nmr.mgh.harvard.edu/fswiki/mri_deface). Clinical data is used for publication with the permission of the patient.

**References**

Griffis, J. C., Allendorfer, J. B., & Szaflarski, J. P. (2016). Voxel-based Gaussian naïve Bayes classification of ischemic stroke lesions in individual T1-weighted MRI scans. *Journal of Neuroscience Methods*, *257*, 97–108. https://doi.org/10.1016/j.jneumeth.2015.09.019
